# Authorship Disputes in Scholarly Biomedical Publications and Trust in the Research Institution

**DOI:** 10.5041/RMMJ.10503

**Published:** 2023-07-31

**Authors:** Itamar Ashkenazi, Oded Olsha

**Affiliations:** 1 The Ruth and Bruce Rappaport Faculty of Medicine, Technion–Israel Institute of Technology, Haifa, Israel; 2 General Surgery Department, Rambam Health Care Campus, Haifa, Israel; 3 General Surgery Department [Emeritus], Shaare Zedek Medical Center, Jerusalem, Israel; 4 Hadassah Faculty of Medicine [Emeritus], Hebrew University of Jerusalem, Jerusalem, Israel

**Keywords:** Author dispute, authorship criteria, CRediT, ghost authorship, gift authorship, ICMJE, publication ethics, scholarly publications

## Abstract

**Introduction:**

When authorship disputes arise in academic publishing, research institutions may be asked to investigate the circumstances. We evaluated the association between the prevalence of misattributed authorship and trust in the institution involved.

**Methods:**

We measured trust using a newly validated Opinion on the Institution’s Research and Publication Values (OIRPV) scale (range 1–4). Mayer and Davies’ Organizational Trust for Management Instrument served as control. Association between publication misconduct, gender, institution type, policies, and OIRPV-derived Trust Scores were evaluated.

**Results:**

A total of 197 responses were analyzed. Increased reporting of authorship misconduct, such as gift authorship, author displacement within the authors’ order on the byline, and ghost authorship, were associated with low Trust Scores (*P*<0.001). Respondents from institutions whose administration had made known (declared or published) their policy on authorship in academic publications awarded the highest Trust Scores (median 3.06, interquartile range 2.25 to 3.56). Only 17.8% favored their administration as the best authority to investigate authorship dispute honestly. Of those who did not list the administration as their preferred option for resolving disputes, 58.6% (95/162) provided a Trust Score <2.5, which conveys mistrust in the institution.

**Conclusions:**

Increased reporting of publication misconducts such as gift authorship, author displacement within the order of the authors’ byline, and ghost authorship was associated with lower Trust Scores in the research institutions. Institutions that made their policies known were awarded the highest Trust Scores. Our results question whether the research institutions’ administrations are the appropriate authority for clarifying author disputes in all cases.

## INTRODUCTION

Author dispute is one of the most common complaints encountered in academic publishing.^1^ The main reason for these disputes is the claim by one or more individuals against the principal author that they were either not acknowledged (i.e. ghost authorship) or were placed on the authors’ byline in a place that does not represent their relative contribution (i.e. author displacement).

Journal editors are empowered to set authorship criteria for their journals. These are commonly based on criteria set by international organizations, such as the International Committee of Medical Journal Editors (ICMJE), the World Association of Medical Editors, and the Council of Scientific Editors, that define significant contributions.^2^–^5^ The CRediT taxonomy is used to explain particular contributions made by individual authors.^6^ Though a certain threshold of contribution is necessary to determine who should be recognized as an author of a scientific manuscript and who should not, the ICMJE warns against using this threshold to disqualify individuals whose contribution to the idea or design of the work or the collection, analysis, or interpretation of the data was significant but who were denied the opportunity to fulfill the other criteria by the main author.^2^

Lack of adherence to authorship criteria significantly contributes to quarrels between authors. Adding names to the final list of authors whose contribution to the article is in doubt is a common phenomenon.^7^ Such misattributed authorship has many names (honorific authorship, guest authorship, gift authorship, etc.). Adding these names to the author list may contribute to the displacement of legitimate authors within the authors’ byline and even make them disappear altogether.

When authorship problems arise and cannot be resolved directly among the authors, international organizations recommend contacting the institution’s administration where the research was performed to clarify the events leading to the complaint.^2^,^5^,^8^ This is based on the assumption that the institution’s administrators will have the best access to investigate the circumstances.

Although research institutions may claim to support proper academic conduct, assuming that the institutions are the appropriate authority for clarifying conflicts between researchers does not consider additional possible competing interests. The institutions where the research was conducted may not be interested in investigating since revealing the truth may cause harm to the institutions’ reputation. Furthermore, events of an unethical nature may be more common in institutions whose administration does not promote a culture that fosters academic integrity in research and publication. If either of these concerns is considered genuine, the researchers will not trust their institution to investigate a case of author dispute.

To measure trust in the administration of the institution where research is performed, we developed the Opinion on the Institution’s Research and Publication Values (OIRPV) scale. A Trust Score is derived from the degree of agreement respondents had with the OIRPV scale’s nine statements. The main objective of this study was to evaluate the association of misattributed authorship with a low Trust Score. Secondary objectives included assessing whether other demographic and institutional issues are associated with a low Trust Score.

## METHODS

The study was approved by the institutional research ethics committee at Rambam Health Care Campus (protocol RMB-22-0124). The study design and reporting were done in accordance with the Checklist for Reporting Results of Internet E-Surveys (CHERRIES)^9^ (see Supplemental Material).

### Description of Survey Items

The survey was primarily composed of multiple-choice questions, five of which were open-ended.

The survey questions were aimed at gathering five sets of data. The first set (questions 1–6) related to obtaining data about the respondents’ background (world region, gender), main area of research (biomedical, other), and experience as either a primary author or coauthor of a biomedical publication. The second set (questions 13 and 17) defined the institution where most of the research work was done in the last 3–10 years and whether the institution had made known its policy on authorship in academic publications. The third set (questions 14–16) collected respondents’ opinions on misattributed authorship, compliance with the ICMJE criteria, and the best authority to deal with an author dispute. The fourth set (questions 7–11) was aimed at gathering information about the respondents’ experience with misattributed authorship, author displacement, and ghost authorship in their research institutions. The experience of respondents was stratified into four categories: rare (0%–5% of publications); uncommon (5.1%–20% of publications); common (20.1%–60% of publications); and very common (over 60% of publications). The fifth set was aimed at evaluating participant Trust Scores (question 12) and scores derived from the Organization Trust Management (TM) scale developed by Mayer and Davis for validation purposes (questions 18 and 19).^10^ Questions 12, 18, and 19, were comprised of 9, 11, and 10 statements, respectively, each of which needed to be graded by the participant. The statements in question 9 were used to calculate the OIRPV Trust Score and the statements in questions 18 and 19 were used for calculating the TM score.^10^ A final question gave participants the option of having their answers included or withdrawn from the survey.

A copy of the survey can be accessed in the Supplementary Material.

### OIRPV Survey Validation Process

The survey, including the OIRPV scale statements, underwent content validation by two experts in publication ethics. The survey draft was disseminated through SurveyMonkey (SVMK Inc., San Mateo, CA, USA) in May and June 2022. Sixty-one respondents participated in the validation phase. Content validity was assessed by analyzing the variation between the respondents’ Trust Scores and the TM scores. While the OIRPV scale statements specifically address respondent opinions on their institutions’ research and publication integrity values, the TM scale statements developed by Mayer and Davies have a broader focus. The latter are designed to asses trust between the trustor and trustee in any organizational framework.

Briefly, the OIRPV scale includes nine statements that are graded according to the degree of agreement or disagreement (strongly disagree, 1 point; somewhat disagree, 2 points; somewhat agree, 3 points; strongly agree, 4 points). The TM scale developed by Mayer and Davis (adapted for this study with permission from the authors and the American Psychological Association) has been previously validated.^10^,^11^ It includes 21 statements that are also graded according to the degree of agreement or disagreement (disagree strongly, 1 point; disagree, 2 points; neither agree nor disagree, 3 points; agree, 4 points; agree strongly, 5 points). Both the OIRPV and TM scales contain negatively and positively worded statements. Before calculating the scores, points awarded to negatively worded statements are re-coded to conform to the positively worded statements. The sum of the points divided by the respective number of statements provides the OIRPV’s Trust Score and TM scores for each individual respondent. An OIRPV Trust Score <2.5 conveys mistrust in the administration, whereas for the TM it is indicated by a score of <3. Note that the OIRPV scale includes an even number of options to grade each statement, forcing discrimination between those who agree and those who disagree with each statement, whereas the TM scale also includes a neutral option of “neither agree nor disagree.”

There was a positive association (Spearman’s rho=0.73; *P*<0.001) between the OIRPV’s Trust Scores and TM scores provided by respondents in the validation phase. The OIRPV scale was further evaluated, resulting in a Cronbach’s alpha value of 0.88, confirming internal consistency. Minor adaptations were made following comments made by the validation participants. Beyond the validation phase, the answers provided by these 61 respondents were not included in the final analysis.

### OIRPV Survey Dissemination

With the aim of targeting authors of biomedical research, the final version of this open survey was distributed through Twitter (Elisabeth Bik’s @MicrobiomDigest), the International Assessment Group of Online Surgical Education network, and by email to 2333 randomly picked coauthors of articles published in SCImago’s list of miscellaneous medical publications for 2019.^12^ A feature in SurveyMonkey was selected that enables participants to complete the survey just once from the same device. To preserve anonymity, no IP addresses were collected. Scrolling through questions, going back and forth, and editing responses were enabled.

The message posted on Twitter and emails contained introductory information and an invitation to participate. The introduction ended with a link to the survey questions listed in SurveyMonkey. The survey introduction made it known that participation was voluntary and that identifiers would not be captured. Participants were asked to respond to all the questions. Other than gratitude, no rewards were offered. The institution’s research ethics committee exempted this study from the need to receive a priori written consent for participation.

### Data Analysis

Responses were collected over four months (mid-September 2022 to mid-January 2023). Individual responses were entered into an Excel database (Microsoft Excel ©2010 Microsoft Corporation, Redmond, WA, USA). As a standard quality check to reduce the odds of including a survey completed by the same person but on a different device, all surveys were compared to each other for uniqueness; none of the included surveys were identical.

Included in this analysis were respondents who agreed to have their answers included and who fully graded the OIRPV scale statements. The resulting Trust Score served as the dependent variable. Since this is the first study to evaluate the Trust Score following the smaller validation study, we re-evaluated the internal consistency of the OIRPV statements with Cronbach’s alpha. Contingency tables were analyzed for specific questions with either two-sided Fisher’s exact test or chi-square for independence. The distribution of Trust Scores within the subgroups was not always normal. Hence, the non-parametric tests (Mann–Whitney, Kruskal–Wallis) were used to evaluate differences between median Trust Scores of the different subgroups. The correlational approach was used to assess whether trends were real for ordinal data with more than two answers (Spearman’s rho). Data were analyzed with GraphPad Prism version 6.00 for Windows (GraphPad Software, La Jolla, CA, USA) and IBM SPSS Statistics for Windows, Version 28.0 (released 2021; IBM Corp., Armonk, NY, USA). The frequencies of the different responses were presented as rates and 95% confidence intervals (95% CI) rounded to the nearest hundredth. Medians and interquartile ranges were rounded to the nearest decimal. Significant *P* values (*P*<0.05) were rounded to the nearest thousandth, while non-significant ones were rounded to the nearest hundredth.

## RESULTS

### Study Participants

The survey was accessed by 209 subjects. After removing 6 participants who opted for their answers to be removed, 203 responses were then evaluated. Responses from 6 subjects were excluded due to incomplete answers that prevented computing their OIRPV’s Trust Score (the main endpoint). A total of 197 subjects were therefore evaluated for this study. Internal consistency was again confirmed by a Cronbach’s alpha value of 0.90.

### Trust Scores Across Study Participants

**Figure 1:**
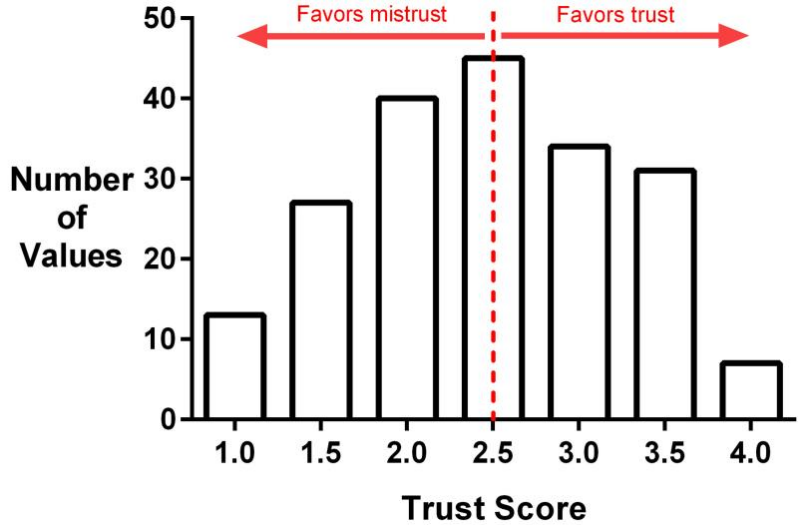
Histogram of the Distribution of Trust Scores. Trust Scores were calculated by adding the degree of agreement with each statement in question 12 (scored between 1 [strongly disagree] and 4 [strongly agree]) divided by the number of statements. A Trust Score <2.5 conveys mistrust in the administration.

Figure 1 shows the distribution of Trust Scores for the 197 responses (median Trust Score 2.44, IQR 1.78 to 3.0). Of these, 46.2% (91/197) respondents favored trust in the administration, while 53.8% (106/197) favored mistrust.

Table 1 presents Trust Scores measured for different attributes of the respondents, their institutions, and their opinions on misattributed authorship and fulfilling the ICMJE criteria. Though women’s responses favored mistrust compared to those of men, these differences did not achieve significance. Trust Score was positively associated with increasing experience as a coauthor and as main author of a biomedical publication. Trust Scores were highest for research institutes and lowest for hospitals. However, these differences did not attain significance. Only 24.4% (48/197) reported that their institution’s administration had made known (declared and/or published) its policy on authorship in academic publications. Trust Scores of respondents whose institutions had declared their policies were significantly higher when compared to respondents who were either unaware or answered their institutions did not make their policies known. No differences in Trust Scores were observed in respondents who thought misattributed authorship should always be condemned compared to those who believed that there is place for this practice under certain circumstances. Neither did Trust Scores differ among respondents with different opinions on the minimum number of the ICMJE criteria needed for authorship.

**Table 1:** Association of Variables with Trust Score.

Variable (*n* of Respondents)		Median Trust Score (Interquartile Range)	*P* Value
**Respondent Backgrounds**			

Gender	Women (64)	2.33 (1.78 to 2.78)	Women vs Men 0.09
	Men (124)	2.56 (1.89 to 3.19)	
	Not disclosed (9)	2.44 (1.78 to 2.72)	

World region	Africa (6)	2.72 (2.42 to 3.58)	0.34
	Asia (21)	2.44 (2.11 to 3.39)	
	Europe (76)	2.44 (1.67 to 2.97)	
	North America (65)	2.33 (2.00 to 2.78)	
	South and Central America (8)	2.94 (1.92 to 3.44)	
	Oceania (14)	2.61 (1.97 to 3.36)	

Main research area	Biomedical (164)	2.44 (1.89 to 3.11)	0.46
	Other (33)	2.33 (1.78 to 2.89)	

Experience as an author/coauthor of biomedical publication	0–5 publications (40)	2.17 (1.56 to 2.78)	<0.001
	6–10 publications (26)	2.22 (1.78 to 2.72)	
	11–30 publications (47)	2.44 (1.67 to 2.78)	
	Over 30 publications (84)	2.67 (2.11 to 3.33)	

Experience as main author of biomedical publication	0–5 publications (82)	2.22 (1.67 to 2.78)	<0.001
	6–10 publications (30)	2.39 (1.94 to 3.00)	
	11–30 publications (39)	2.44 (1.67 to 2.89)	
	Over 30 publications (46)	2.83 (2.33 to 3.47)	

**Institutional Attributes**			

Institution where research was done	University/college/other educational (139)	2.44 (2.00 to 3.11)	0.09
	Research institute (23)	2.67 (1.78 to 3.33)	
	Hospital (33)	2.11 (1.44 to 2.83)	

Institution’s ethics policy on authorship declared	Yes (48)	3.06 (2.25 to 3.56)	<0.001
	No (86)	2.22 (1.67 to 2.67)	
	I do not know (63)	2.33 (2.00 to 3.11)	

**Respondent Opinions on Fulfilling the ICMJE Criteria and Misattributed Authorship**			

Opinion on misattributed authorship	Should be condemned in all circumstances (133)	2.33 (1.78 to 3.00)	0.24
	There is place for this in certain circumstances (64)	2.56 (2.00 to 3.19)	

Number of ICMJE criteria necessary to become an author	1 (30)	2.50 (1.89 to 3.14)	0.97
	2 (39)	2.44 (2.22 to 2.78)	
	3 (51)	2.44 (1.78 to 3.00)	
	4 (77)	2.44 (1.66 to 3.06)	

Trust Scores were calculated by adding the degree of agreement with each statement in question 12 (scored between 1 [strongly disagree] and 4 [strongly agree]) divided by the number of statements. A Trust Score <2.5 conveys mistrust in the administration.

* Missing data: World region, 7 respondents; Institution where research was done, 2 respondents.

### Association of Misattributed Authorship with Low Trust Scores

Table 2 presents the association between different types of misattributed authorship and the Trust Score. Whether perceived as prevalent in the institution’s publications or personally experienced, increasing rates of misattributed authorship were associated with lower Trust Scores.

**Table 2:** The Correlation Between Perceived Misconduct or Actual Experience of Misconduct in Publication (Survey Questions 7–11) and Trust Score in 197 Respondents.

Question/Possible Answers	*N*	Rate (95% Confidence Interval)	Median Trust Score (Interquartile Range)	ρ (*P* Value)
In your opinion, in your workplace/research environment, how often were academic manuscripts published in which individuals were added to the list of authors, though they did not contribute significantly to the work being published?*				
Rare (0% to 5% of publications)	38	0.19 (0.14 to 0.26)	3.28 (2.64 to 3.67)	ρ =−0.52 (<0.001)
Uncommon (5.1% to 20% of publications)	61	0.31 (0.25 to 0.38)	2.78 (2.28 to 3.11)	
Common (20.1% to 60% of publications)	64	0.33 (0.26 to 0.40)	2.22 (1.58 to 2.67)	
Very common (over 60% of publications)	33	0.17 (0.12 to 0.23)	2.00 (1.33 to 2.33)	

In your opinion, in your workplace/research environment, how often were co-authors displaced from their appropriate place in the list of authors?				
Rare (0% to 5% of publications)	82	0.42 (0.35 to 0.49)	2.78 (2.22 to 3.36)	ρ =−0.37 (<0.001)
Uncommon (5.1% to 20% of publications)	59	0.30 (0.24 to 0.37)	2.44 (1.78 to 2.89)	
Common (20.1% to 60% of publications)	38	0.19 (0.14 to 0.26)	2.06 (1.64 to 2.67)	
Very common (over 60% of publications)	18	0.09 (0.06 to 0.14)	2.22 (1.39 to 2.39)	

In your opinion, in your workplace/research environment, how often were individuals who significantly contributed to the academic work being published not acknowledged at all as authors in the final publication?				
Rare (0% to 5% of publications)	128	0.65 (0.58 to 0.72)	2.67 (2.11 to 3.31)	ρ =−0.27 (<0.001)
Uncommon (5.1% to 20% of publications)	47	0.24 (0.18 to 0.30)	2.11 (1.56 to 2.56)	
Common (20.1% to 60% of publications)	17	0.09 (0.05 to 0.14)	2.33 (2.00 to 2.83)	
Very common (over 60% of publications)	5	0.03 (0.01 to 0.06)	2.00 (1.33 to 2.28)	

How many times have you personally been an author, co-author, contributed to, or involved in a manuscript in which Gift Authorship was awarded?				
Rare (0% to 5% of publications)	116	0.59 (0.52 to 0.66)	2.67 (2.22 to 3.33)	ρ =−0.40 (<0.001)
Uncommon (5.1% to 20% of publications)	36	0.18 (0.13 to 0.24)	2.33 (1.89 to 2.86)	
Common (20.1% to 60% of publications)	28	0.14 (0.10 to 0.20)	1.89 (1.47 to 2.22)	
Very common (over 60% of publications)	17	0.09 (0.05 to 0.14)	2.00 (1.28 to 2.44)	

How many times have you personally been an author, co-author, contributed to, or involved in a manuscript in which individuals who contributed significantly to the academic work being published were either displaced within the authors’ byline or not acknowledged at all?				
Rare (0% to 5% of publications)	137	0.70 (0.63 to 0.76)	2.67 (2.11 to 3.33)	ρ =−0.35 (<0.001)
Uncommon (5.1% to 20% of publications)	36	0.18 (0.13 to 0.24)	2.33 (1.67 to 2.78)	
Common (20.1% to 60% of publications)	19	0.10 (0.06 to 0.15)	2.00 (1.56 to 2.22)	
Very common (over 60% of publications)	5	0.03 (0.01 to 0.06)	1.44 (1.22 to 2.11)	

Trust Scores were calculated by adding the degree of agreement with each statement in question 12 (scored between 1 [strongly disagree] and 4 [strongly agree]) divided by the number of statements. A Trust Score <2.5 conveys mistrust in the administration.

* Missing data, 1 respondent.

### Influence of Institutional Policies and Other Variables on Misattributed Authorship Rates

**Figure 2:**
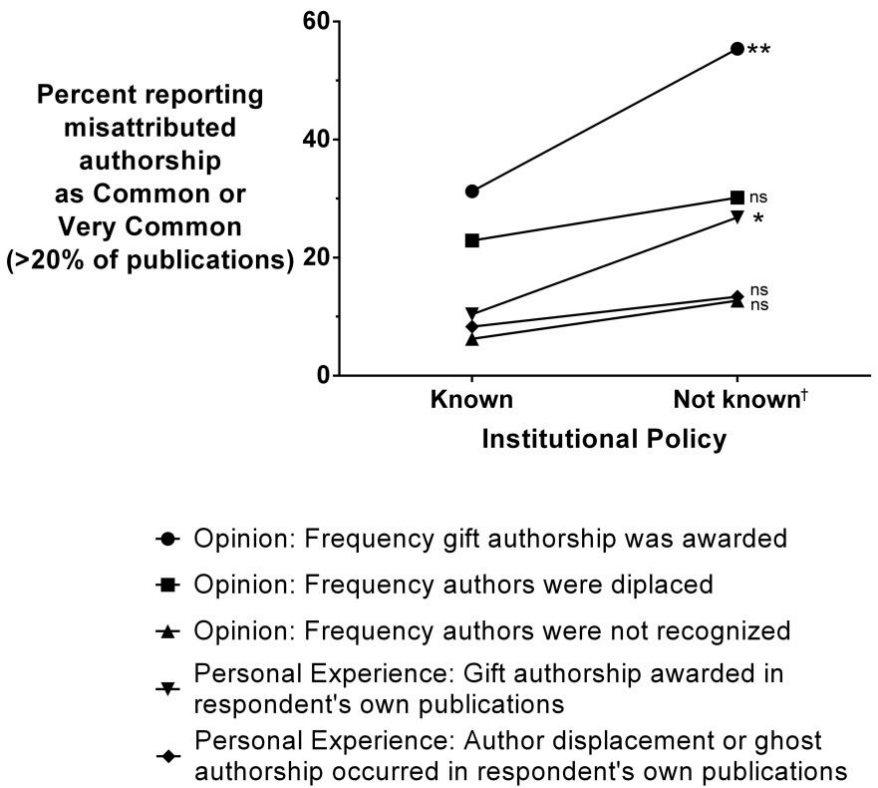
Association of Misattributed Authorship Rated as Common or Very Common with Whether or Not an Institution Made Its Authorship Policy Known. ns, nonsignificant. * <0.05; ** <0.01; ^†^ either did not make known, or unknown.

Since Trust Scores were highest from respondents whose institutions declared their policies, we examined whether there were differences in the reporting of different types of misconduct regarding authorship between respondents’ institutions that did and did not make their policy known (Figure 2). All types of misattributed authorship were reported less by respondents working in institutions that made their policies on authorship in academic publications known. However, only “gift authorship” reached significance. Differences in the reporting of different types of misattributed authorship between men and women, and those working in hospitals as compared to other institutions, are presented in Supplemental Figures 1 and 2.

### Best Authority to Deal with Authorship Disputes Honestly

**Figure 3:**
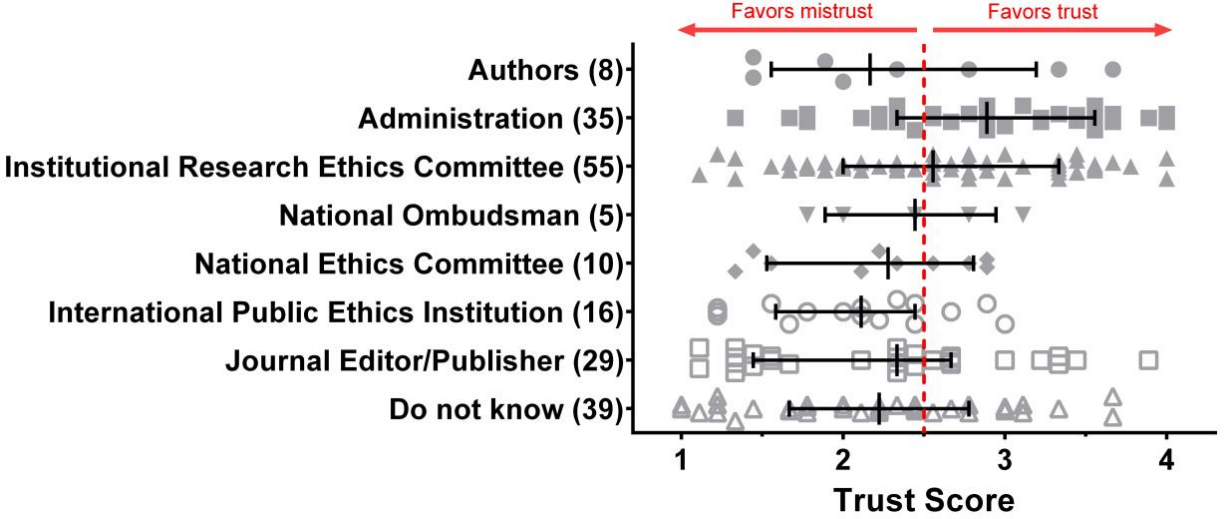
Survey Answers for Best Authority to Deal with an Author Dispute and the Corresponding Trust Scores for Their Institutions. The numbers of respondents are indicated in parenthesis. Bars indicate median score and interquartile ranges. Trust Scores were calculated by adding the degree of agreement with each statement in question 12 (scored between 1 [strongly disagree] and 4 [strongly agree]) divided by the number of statements. A Trust Score <2.5 conveys mistrust in the administration.

Survey participants were asked which authority they thought would be best to honestly deal with a dispute if they were personally involved in an author dispute. The responses and their association with Trust Score are presented in Figure 3.

Only 17.8% (35/197) favored their administration. Of those who did not list the administration as their preferred option for resolving disputes, 58.6% (95/162) provided a Trust Score <2.5, which conveys mistrust in the administration. Of note, eight of those who responded “others” indicated that the authors themselves should deal with the conflict.

## DISCUSSION

The main finding of this survey is that increased reporting of authorship misconduct, such as gift authorship, author displacement, and ghost authorship, were associated with low Trust Scores. The highest Trust Scores were given by respondents whose administration had made known (declared or published) their policy on authorship in academic publications. In these institutions, the percentage reporting gift authorship was lower compared to institutions that did not make their policies known. Lower rates of displaced authorship and ghost authorship reported by these respondents did not attain significance.

When presented with a situation where they would be personally involved in an author dispute, only 17.8% favored their administration as the best authority to deal with this dispute honestly. Over half of the other respondents provided the administration with a low Trust Score. These findings indicate that author disputes that the authors cannot resolve should not be automatically referred to the authors’ institutions. Such a referral should be made only after the journal editors have evaluated the trust that the feuding authors have in their institutions’ administrations to resolve the dispute honestly.

The high prevalence of low scores awarded to institutional administration on ethical issues related to research and academic publications is disappointing. This includes low Trust Scores afforded to hospitals where biomedical research is done. Even though relatively few of those approached participated in this study and selection bias is a possibility, the common assertion, which grants trust in the administration to resolve authorship issues and other misconduct, is at best, unfounded.

Certain limitations should be taken into account. Only a minority of those approached participated in this study, and, as discussed above, this could lead to selection bias. Thus, caution should be exercised in generalizing the rates of different options reported in this study. Nevertheless, regardless how many chose to participate, relatively equal numbers of participants were recruited who either favored trust or favored mistrust in their research institution’s administration. Significant associations between the Trust Score and several of the variables included in this study should not be ruled out. Whether the high rate of those expressing mistrust in their institution noted in this study reliably reflects the prevailing situation can only be assessed in a study with randomly picked participants and higher recruitment rates. Larger samples would be needed to investigate whether low Trusts Scores are more prevalent for women and those investigators who work in hospitals.

Another limitation of this study was that we concentrated on author displacement and ghost authorship in evaluating trust in the research institution’s values. However, other situations may lead to authorship disputes, which were not directly evaluated in this study. These include cases in which coauthors remove themselves from studies because they disagree with the interpretation or presentation of the data embraced by the principal investigator. Whether this situation is common has yet to be explored.

Research regarding publication ethics is mainly centered on the frequency of misconduct declared. The methodology described provides a tool to analyze the function of institution administrations in terms of publication ethics by evaluating the association between OIRPV-derived Trust Scores and rates of misbehavior declared. Although this study focused on biomedical research, there is a place to evaluate this methodology in other academic areas, such as social sciences and the humanities.

International organizations are responsible for defining contributions worthy of authorship, what constitutes ethical behavior, and what is considered misconduct.2,4,5,13 Beyond that, the recommended approach to addressing authorship issues is multifaceted. Institutions involved in research play a central role in dealing with these issues.14 These institutions are expected to set an example and create a culture that fosters research and publication integrity. Beyond education and training, leaders of research institutions must protect ethical scientific practice by being open to admitting and addressing the possibility that misconduct occurs under their remit. Still, authorship issues remain abundant, and some end up as complaints termed “author disputes.” We argue that the issues underlining these disputes are much larger than just a personal quarrel between individual contributors. If there is extensive mistrust in research institutions, it cannot be ruled out that, in such an environment, many contributors will simply give up on their authorship rights, and only the minority of cases end up as complaints. Where such mistrust is pervasive, this problem is severely underestimated, both in magnitude and extent.

## CONCLUSION

This study found that both high levels of reported misattributed authorship and lack of clarity about institutional authorship policies were associated with low Trust Scores. Whenever author disputes arise, if the authors cannot resolve their conflict, journal editors and publishers should not assume that the authors’ institution is the appropriate authority for clarifying conflicts between researchers.

## Supplementary Information



## Data Availability

Data will be made available by Itamar Ashkenazi.*

## Abbreviations

ICMJE: International Committee of Medical Journal Editors
OIRPV: opinion on the institution’s research and publication values
TM: organizational trust for management
