# Supplementary material for: Authorship Disputes in Scholarly Biomedical Publications and Trust in the Research Institution
Source: Rambam Maimonides Med J. 2023 Jul 31;14(3):e0015. doi: 10.5041/RMMJ.10503 (PMC10393470; doi:10.5041/RMMJ.10503)
Supplement: Supplementary file 1 [file rmmj-14-3-e0015AM.docx]

This appendix has been provided by the authors for the benefit of readers

Supplement to Authorship Disputes in Scholarly Biomedical Publications and Trust in the Research Institution

Ashkenazi I, Olsha O. Authorship Disputes in Scholarly Biomedical Publications and Trust in the Research Institution. Rambam Maimonides Med J 2023;14(3): e0015. doi:10.5041/RMMJ.10503

The supplementary material provided by the authors is as listed below.

# CONTENTS

[**Supplement 1:**  Copy of Survey on Misattributed Authorship 2](#_Toc141181620)

[**Supplement 2:**The Association of Misattributed Authorship Rated as Common or Very Common With Gender and Type of Research Institute 8](#_Toc141181624)

[**Supplement 3:**  Checklist for Reporting Results of Internet E-Surveys (CHERRIES) 10](#_Toc141181625)

# Supplement 1: Copy of Survey on Misattributed Authorship

*Note: An updated version of the survey will continue collecting answers at* [*https://forms.gle/amR8YDXQQuczBj9M9*](https://forms.gle/amR8YDXQQuczBj9M9)

Welcome to My Survey on Misattributed Authorship and Author Dispute

Dear Colleague,

I invite you to participate in this survey about misattributed authorship and author disputes in academic publications. This survey is about the following subjects:

Misattributed authorship encompasses two situations: (1) individuals who are granted authorship in a manuscript while their contribution was minimal, if at all; (2) individuals who did contribute signiﬁcantly but are not appropriately recognized for their eﬀort within the authors’ byline, if at all.

Author dispute commonly occurs when certain individuals are dissatisﬁed with the place allotted to them within the authors’ byline. It may be that their place within the authors’ byline does not represent, in their opinion, their contribution to the manuscript when compared to other authors. It may be that they do not appear at all within the list of authors (i.e., ghost authors) even though they are of the opinion that their contribution was signiﬁcant and they deserved to be included.

Workplace environment - In this survey, we also wish to explore how the administration of your workplace/research environment aﬀects your experience with honest research and honest research publication. If you have worked in several places, please choose the one place where you have done most of your research work during the last 3-10 years.

## The survey

The request to participate in this survey will be sent to several thousand potential participants. The survey comprises 21 questions. Three of these questions contain sentences for which you will need to score your degree of agreement with the statement. It should take about 8-10 minutes to answer. Answers will be collected and published without identiﬁers other than world region, gender identity, and profession. This survey was authorized by a research ethics committee (protocol RMB-0124-22)

## Participation in this survey

Participation in this survey is voluntary. The last question asks you to authorize your voluntary participation. You may discontinue your participation at any time by choosing the possibility “I wish to discontinue my participation - please remove my answers from the ﬁnal analysis”. Since no identiﬁers will be collected, we will not be able to discontinue participation once the survey has been submitted. If you have received the request to participate from diﬀerent sources or on multiple devices, please ﬁll out this survey only once. Fully answering the survey questions and scoring the statements is needed to help analyze the answers. Other than our gratitude, we cannot compensate participants. If the results of this survey are published, this will be done without identiﬁers. Comments may be sent directly to my email.

Thank you,

Itamar Ashkenazi, Rambam Health Care Campus, Technion Institute of Technology (personal email: i_ashkenazi@yahoo.com)

# Survey on Misattributed Authorship

|  | Please indicate the region where most of your work/research was performed in the last 10 years: | | | | | | | | | | |
| --- | --- | --- | --- | --- | --- | --- | --- | --- | --- | --- | --- |
|  | 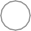 | North America (Mexico, USA, Canada) | | | | |  | | | | |
|  | 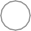 | South and Central America | | | | |  | | | | |
|  | 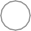 | Europe | | | | |  | | | | |
|  | 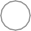 | Africa | | | | |  | | | | |
|  | 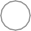 | Asia | | | | |  | | | | |
|  | 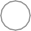 | Oceania | | | | |  | | | | |
|  | 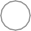 | Optional (list country’s name): | | |  | | | | | | |
|  |  |  | | | | |  | | | | |
|  | With which gender do you most identify? Please choose one option. | | | | | | | | | | |
|  | 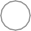 | Woman | | | | |  | | | | |
|  | 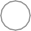 | Man | | | | |  | | | | |
|  | 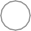 | Non-binary or Gender diverse | | | | |  | | | | |
|  | 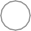 | Prefer not to disclose | | | | |  | | | | |
|  |  |  | | | | |  | | | | |
|  | Please indicate your main area of research? | | | | | | | | | | |
|  | 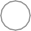 | Biomedical research | | | | |  | | | | |
|  | 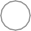 | Other (list): | |  | | | | | | | |
|  |  |  | | | | |  | | | | |
|  | Please indicate your specialty: | | | | | | | | | | |
|  | 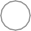 | Researcher (not a physician) | | | | |  | | | | |
|  | 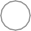 | Medical (non-surgical) specialty, mainly adults | | | | |  | | | | |
|  | 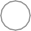 | Medical (non-surgical) specialty, mainly children | | | | |  | | | | |
|  | 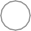 | Surgical specialty, mainly adults | | | | |  | | | | |
|  | 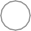 | Surgical specialty, mainly children | | | | |  | | | | |
|  | 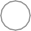 | Other (please specify): | |  | | | | | | | |
|  |  |  | | | | |  | | | | |
|  | How many times have you been the author/co-author of a biomedical publication? | | | | | | | | | | |
|  | 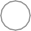 | 0-5 | | | | |  | | | | |
|  | 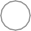 | 6-10 | | | | |  | | | | |
|  | 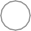 | 11-30 | | | | |  | | | | |
|  | 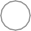 | 31 and above | | | | |  | | | | |
|  |  |  | | | | |  | | | | |
|  | How many times have you been main author of a biomedical publication? | | | | | | | | | | |
|  | 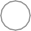 | 0-5 | | | | |  | | | | |
|  | 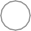 | 6-10 | | | | |  | | | | |
|  | 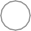 | 11-30 | | | | |  | | | | |
|  | 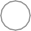 | 31 and above | | | | |  | | | | |
|  |  |  | | | | |  | | | | |
|  | In your opinion, in your workplace/research environment, how often were academic manuscripts published in which individuals were added to the list of authors, though they did not contribute signiﬁcantly to the work being published? | | | | | | | | | | |
|  | 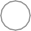 | Rare (0-5% of publications) | | | | |  | | | | |
|  | 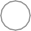 | Uncommon (5.1 to 20% of publications) | | | | |  | | | | |
|  | 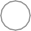 | Common (20.1% to 60% of publications) | | | | |  | | | | |
|  | 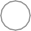 | Very common (over 60% of publications) | | | | |  | | | | |
|  |  |  | | | | |  | | | | |
|  | In your opinion, in your workplace/research environment, how often were co-authors displaced from their appropriate place in the list of authors? | | | | | | | | | | |
|  | 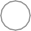 | Rare (0-5% of publications) | | | | |  | | | | |
|  | 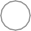 | Uncommon (5.1 to 20% of publications) | | | | |  | | | | |
|  | 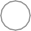 | Common (20.1% to 60% of publications) | | | | |  | | | | |
|  | 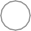 | Very common (over 60% of publications) | | | | |  | | | | |
|  |  |  | | | | |  | | | | |
|  | In your opinion, in your workplace/research environment, how often were individuals who signiﬁcantly contributed to the academic work being published not acknowledged at all as authors in the ﬁnal publication? | | | | | | | | | | |
|  | 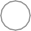 | Rare (0-5% of publications) | | | | |  | | | | |
|  | 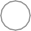 | Uncommon (5.1 to 20% of publications) | | | | |  | | | | |
|  | 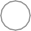 | Common (20.1% to 60% of publications) | | | | |  | | | | |
|  | 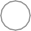 | Very common (over 60% of publications) | | | | |  | | | | |
|  |  |  | | | | |  | | | | |
|  | How many times have you personally been an author, co-author, contributed to, or involved in a manuscript in which “Gift Authorship” was awarded? | | | | | | | | | | |
|  | 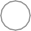 | Rare (0-5% of publications) | | | | |  | | | | |
|  | 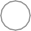 | Uncommon (5.1 to 20% of publications) | | | | |  | | | | |
|  | 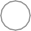 | Common (20.1% to 60% of publications) | | | | |  | | | | |
|  | 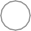 | Very common (over 60% of publications) | | | | |  | | | | |
|  |  |  | | | | |  | | | | |
|  | How many times have you personally been an author, co-author, contributed to, or involved in a manuscript in which individuals who contributed signiﬁcantly to the academic work being published were either displaced within the authors’ byline or not acknowledged at all? | | | | | | | | | | |
|  | 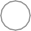 | Rare (0-5% of publications) | | | | |  | | | | |
|  | 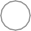 | Uncommon (5.1 to 20% of publications) | | | | |  | | | | |
|  | 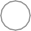 | Common (20.1% to 60% of publications) | | | | |  | | | | |
|  | 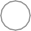 | Very common (over 60% of publications) | | | | |  | | | | |
|  |  |  | | | | |  | | | | |
|  | Please indicate if you strongly disagree (1), somewhat disagree (2), somewhat agree (3), or strongly agree (4) with the following nine statements concerning the administration of the institution where you have done most of your research work in the last 3-10 years: | | | | | | | | | | |
|  | | | **(1) Strongly disagree** | | **(2) Somewhat disagree** | **(3) Somewhat agree** | | **(4) Strongly agree** | | |  |
| The administration take active steps to promote honest research. | | | 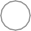 | | 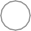 | 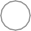 | | 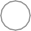 | | |  |
| The administration take active steps to condemn misattributed authorship. | | | 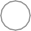 | | 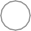 | 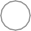 | | 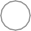 | | |  |
| The administration actively take an interest in your rights as contributors in research and publication. | | | 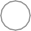 | | 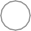 | 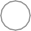 | | 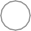 | | |  |
| If you were involved in a case of author dispute with another author within your institution, you would want the institution’s administration to be involved in resolving the dispute. | | | 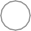 | | 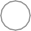 | 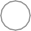 | | 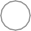 | | |  |
| If you were involved in a case of author dispute with another author within your institution, you trust you would be treated fairly by the administration of your institution if they were asked to intervene. | | | 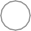 | | 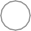 | 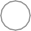 | | 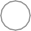 | | |  |
| The leaders of the administration place honest research and/or honest publication above their personal relationships with different researchers in the institutions. | | | 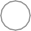 | | 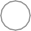 | 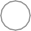 | | 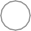 | | |  |
| The administration care about honest research. | | | 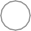 | | 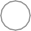 | 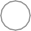 | | 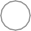 | | |  |
| The administration care about honest publication. | | | 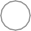 | | 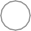 | 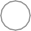 | | 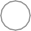 | | |  |
| In case of author disputes, the administration place personal interests before honest research and publication. | | | 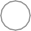 | | 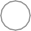 | 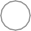 | | 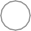 | | |  |
|  | | |  | |  |  | |  | | |  |
|  | Which answer best deﬁnes the institution where you have done most of your research work in the last 3-10 years: | | | | | | | | | | |
|  | 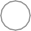 | University | | | | |  | | | | |
|  | 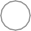 | College | | | | |  | | | | |
|  | 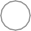 | Research institution | | | | |  | | | | |
|  | 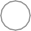 | Hospital | | | | |  | | | | |
|  | 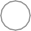 | Other (please specify): | |  | | | | | | | |
|  |  |  | | | | |  | | | | |
|  | If you were personally involved in a situation of an author dispute, who do you think is the best authority to deal with this dispute in an honest way? | | | | | | | | | | |
|  | 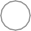 | The journal’s editor | | | | |  | | | | |
|  | 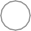 | The journal’s publisher | | | | |  | | | | |
|  | 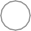 | The administration where you perform your research | | | | |  | | | | |
|  | 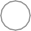 | The institution’s research ethics committee | | | | |  | | | | |
|  | 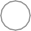 | A national ombudsman | | | | |  | | | | |
|  | 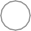 | A national ethics committee | | | | |  | | | | |
|  | 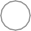 | An international institution that deals with publication ethics | | | | | | | | |  |
|  | 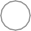 | Do not know | | | | |  | | | | |
|  | 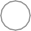 | Other (please specify): | |  | | | | | | | |
|  |  |  | | | | |  | | | | |
|  | The International Committee of Medical Journal Editors (ICMJE) deﬁned four criteria for establishing authorship:  (1) Substantial contributions to the conception or design of the work; or the acquisition, analysis, or interpretation of data for the work;  (2) Drafting the work or revising it critically for important intellectual content;  (3) Final approval of the version to be published;  (4) Agreement to be accountable for all aspects of the work in ensuring that questions related to the accuracy or integrity of any part of the work are appropriately investigated and resolved.  Which of these sentences best represents your opinion concerning meeting the criteria for authorship as indicated by the ICMJE? | | | | | | | | | | |
|  | 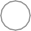 | Authors should comply with at least one of the four criteria | | | | | |  | | | |
|  | 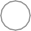 | Authors should comply with at least two of the four criteria | | | | | |  | | | |
|  | 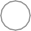 | Authors should comply with at least three of the four criteria | | | | | |  | | | |
|  |  | Authors should comply with all four criteria | | | | | |  | | | |
|  |  |  | | | | |  | | | | |
|  | Which of these sentences best represents your opinion concerning misattributed authorship (gift/ghost/guest)? | | | | | | | | | | |
|  |  | There is place for this practice in certain circumstances | | | | | | | |  | |
|  |  | It should be condemned in all circumstances | | | | | | | |  | |
|  |  |  | | | | |  | | | | |
|  | In your workplace/research environment, has your administration made known (declared and /or published) its policy on authorship in academic publications? | | | | | | | | | | |
|  |  | Yes | | | | |  | | | | |
|  |  | No | | | | |  | | | | |
|  |  | I don’t know | | | | |  | | | | |
|  |  |  | | | | |  | | | | |
|  | ***** The following are general statements that describe what you think about the administration of your workplace/research environment where most of your research was done during the last 3-10 years. For each of the following 11 statements, note the number that best describes how much you agree or disagree with each statement (scale: 1=disagree strongly to 5=agree strongly):  *The above text was followed by the 11 statements on “Ability” and “Benevolence”, which can be found in the Appendix of Mayer and Davis (cited below).* | | | | | | | | | | |
|  |  |  | | | | |  | | | | |
|  | ***** The following are general statements that describe what you think about the administration of your workplace/research environment where most of your research was done during the last 3-10 years. For each of the following 10 statements, note the number that best describes how much you agree or disagree with each statement (scale: 1=disagree strongly to 5=agree strongly):  *The above text was followed by the 10 statements on “Integrity” and “Trust”, which can be found in the Appendix of Mayer and Davis (cited below).* | | | | | | | | | | |
|  |  |  | | | | |  | | | | |
|  | Do you wish to be included or withdrawn from this survey? | | | | | | | | | | |
|  |  | Please include my answers in the final analysis | | | | |  | | | | |
|  |  | I wish to withdraw from this survey – please remove my answers from the final analysis | | | | |  | | | | |
|  |  | |  |  |  |  |  |  |  |  |  |
|  |  |  | | | | |  | | | | |
|  | If you have any comments concerning the survey, please add these below: | | | | | | | | | | |
|  | (Free text field) |  | | | | |  | | | | |
|  |  |  | | | | |  | | | | |
|  |  |  | | | | |  | | | | |

***** Permission was granted to use these questions for the survey only. Copyright ©1999 by the American Psychological Association. Questions were adapted with permission, as follows: The word “management was changed to “administration.” Taken from the Appendix, page 136 of: Mayer RC, Davis JH. The eﬀect of the performance appraisal system on trust for management: A ﬁeld quasi-experiment. J Applied Psych 1999;84:126–36. <https://doi.org/10.1037/0021-9010.84.1.123>. No further reproduction or distribution is permitted without written permission from the American Psychological Association.

# Supplement 2: The Association of Misattributed Authorship Rated as Common or Very Common with Gender and Type of Research Institute

Supplemental Figure 1: Association of Misattributed Authorship Rated as Common or Very Common with Gender.

ns, non-significant.

* <0.05

Supplemental Figure 2: Association of Misattributed Authorship Rated as Common or Very Common with Type of Research Institution (Hospital vs Others).

ns, non-significant.

* <0.05

# Supplement 3: Checklist for Reporting Results of Internet E-Surveys (CHERRIES)

|  | | **Checklist for Reporting Results of Internet E-Surveys (CHERRIES)** |  |
| --- | --- | --- | --- |
| ***Item Category*** | | ***Checklist Item*** | ***Explanation*** |
| **Design** | | | |
|  | Describe survey design | Describe target population, sample frame. Is the sample a convenience sample? (In “open” surveys this is most likely.) | Target population described in the methods section. |
| **IRB (Institutional Review Board) approval and informed consent process** | | | |
|  | IRB approval | Mention whether the study has been approved by an IRB. | IRB approval described in the methods section |
|  | Informed consent | Describe the informed consent process. Where were the participants told the length of time of the survey, which data were stored and where and for how long, who the investigator was, and the purpose of the study? | Described in the methods section. |
|  | Data protection | If any personal information was collected or stored, describe what mechanisms were used to protect unauthorized access. | No personal identifiers collected. Described in the methods section. |
| **Development and pre-testing** | | | |
|  | Development and testing | State how the survey was developed, including whether the usability and technical functionality of the electronic questionnaire had been tested before fielding the questionnaire. | Described in the methods section. |
| **Recruitment process and description of the sample having access to the questionnaire** | | | |
|  | Open survey versus closed survey | An “open survey” is a survey open for each visitor of a site, while a closed survey is only open to a sample which the investigator knows (password-protected survey). | This was an open survey disseminated through Twitter and also sent to specific addresses. Described in the methods section. |
|  | Contact mode | Indicate whether or not the initial contact with the potential participants was made on the Internet. (Investigators may also send out questionnaires by mail and allow for Web-based data entry.) | Sent out by both internet and mail (see methods section) |
|  | Advertising the survey | How/where was the survey announced or advertised? Some examples are offline media (newspapers), or online (mailing lists – If yes, which ones?) or banner ads (Where were these banner ads posted and what did they look like?). It is important to know the wording of the announcement as it will heavily influence who chooses to participate. Ideally the survey announcement should be published as an appendix. | The survey was disseminated through Twitter, through the International Assessment Group of Online Surgical Education network, and by email to 2333 randomly picked coauthors of articles. This is fully described in the methods section. |
| **Survey administration** | | | |
|  | Web/E-mail | State the type of e-survey (eg, one posted on a Web site, or one sent out through e-mail). If it is an e-mail survey, were the responses entered manually into a database, or was there an automatic method for capturing responses? | Responses captured by SurveyMonkey. Individual responses were listed in the database (see methods) |
|  | Context | Describe the Web site (for mailing list/newsgroup) in which the survey was posted. What is the Web site about, who is visiting it, what are visitors normally looking for? Discuss to what degree the content of the Web site could pre-select the sample or influence the results. For example, a survey about vaccination on a anti-immunization Web site will have different results from a Web survey conducted on a government Web site | The twitter account is described in the methods section. |
|  | Mandatory/voluntary | Was it a mandatory survey to be filled in by every visitor who wanted to enter the Web site, or was it a voluntary survey? | Voluntary survey (see methods) |
|  | Incentives | Were any incentives offered (eg, monetary, prizes, or non-monetary incentives such as an offer to provide the survey results)? | NONE offered (see methods and introduction to the survey) |
|  | Time/Date | In what timeframe were the data collected? | Described in the methods section |
|  | Randomization of items or questionnaires | To prevent biases items can be randomized or alternated. | Not performed |
|  | Adaptive questioning | Use adaptive questioning (certain items, or only conditionally displayed based on responses to other items) to reduce number and complexity of the questions. | All responders were asked to answer all the questions. Described in the methods section. |
|  | Number of Items | What was the number of questionnaire items per page? The number of items is an important factor for the completion rate. | The survey is provided with the manuscript and its contents described in the methods section |
|  | Number of screens (pages) | Over how many pages was the questionnaire distributed? The number of items is an important factor for the completion rate. | The number of items is described in the methods section. |
|  | Completeness check | It is technically possible to do consistency or completeness checks before the questionnaire is submitted. Was this done, and if “yes”, how (usually JAVAScript)? An alternative is to check for completeness after the questionnaire has been submitted (and highlight mandatory items). If this has been done, it should be reported. All items should provide a non-response option such as “not applicable” or “rather not say”, and selection of one response option should be enforced. | Since Trust Scores were the endpoint assessed, to be included, the only requisite was to complete the OIRPV statements. This is described in the methods section. Missing data on other variables is mentioned in the tables. |
|  | Review step | State whether respondents were able to review and change their answers (eg, through a Back button or a Review step which displays a summary of the responses and asks the respondents if they are correct). | Yes (see methods) |
| **Response rates** | | | |
|  | Unique site visitor | If you provide view rates or participation rates, you need to define how you determined a unique visitor. There are different techniques available, based on IP addresses or cookies or both. | We did not collect IP addresses or cookies. This was described in the methods section. |
|  | View rate (Ratio of unique survey visitors/unique site visitors) | Requires counting unique visitors to the first page of the survey, divided by the number of unique site visitors (not page views!). It is not unusual to have view rates of less than 0.1 % if the survey is voluntary. | Not performed. The issue of the low number of answers is discussed. |
|  | Participation rate (Ratio of unique visitors who agreed to participate/unique first survey page visitors) | Count the unique number of people who filled in the first survey page (or agreed to participate, for example by checking a checkbox), divided by visitors who visit the first page of the survey (or the informed consents page, if present). This can also be called “recruitment” rate. | The data of how many were excluded was made available at the beginning of the results section. |
|  | Completion rate (Ratio of users who finished the survey/users who agreed to participate) | The number of people submitting the last questionnaire page, divided by the number of people who agreed to participate (or submitted the first survey page). This is only relevant if there is a separate “informed consent” page or if the survey goes over several pages. This is a measure for attrition. Note that “completion” can involve leaving questionnaire items blank. This is not a measure for how completely questionnaires were filled in. (If you need a measure for this, use the word “completeness rate”.) | Inapplicable - This was a one page survey |
| **Preventing multiple entries from the same individual** | | | |
|  | Cookies used | Indicate whether cookies were used to assign a unique user identifier to each client computer. If so, mention the page on which the cookie was set and read, and how long the cookie was valid. Were duplicate entries avoided by preventing users access to the survey twice; or were duplicate database entries having the same user ID eliminated before analysis? In the latter case, which entries were kept for analysis (eg, the first entry or the most recent)? | No IP addresses were collected. However, the program has a unique feature that allows only one response to be collected from one computer. Described above and in the methods. |
|  | IP check | Indicate whether the IP address of the client computer was used to identify potential duplicate entries from the same user. If so, mention the period of time for which no two entries from the same IP address were allowed (eg, 24 hours). Were duplicate entries avoided by preventing users with the same IP address access to the survey twice; or were duplicate database entries having the same IP address within a given period of time eliminated before analysis? If the latter, which entries were kept for analysis (eg, the first entry or the most recent)? | See answer above. |
|  | Log file analysis | Indicate whether other techniques to analyze the log file for identification of multiple entries were used. If so, please describe. | None of the answers submitted by different subjects was identical (methods) |
|  | Registration | In “closed” (non-open) surveys, users need to login first and it is easier to prevent duplicate entries from the same user. Describe how this was done. For example, was the survey never displayed a second time once the user had filled it in, or was the username stored together with the survey results and later eliminated? If the latter, which entries were kept for analysis (eg, the first entry or the most recent)? | Not applicable |
| **Analysis** | | | |
|  | Handling of incomplete questionnaires | Were only completed questionnaires analyzed? Were questionnaires which terminated early (where, for example, users did not go through all questionnaire pages) also analyzed? | The survey answers were all analyzed. All the answers are provided included information on missing answers |
|  | Questionnaires submitted with an atypical timestamp | Some investigators may measure the time people needed to fill in a questionnaire and exclude questionnaires that were submitted too soon. Specify the timeframe that was used as a cut-off point, and describe how this point was determined. | Not performed |
|  | Statistical correction | Indicate whether any methods such as weighting of items or propensity scores have been used to adjust for the non-representative sample; if so, please describe the methods | Not applicable |
